# Supported Lipid Bilayers and the Study of Two-Dimensional Binding Kinetics

**DOI:** 10.3389/fmolb.2022.833123

**Published:** 2022-02-18

**Authors:** Tommy Dam, Manto Chouliara, Victoria Junghans, Peter Jönsson

**Affiliations:** ^1^ Department of Chemistry, Lund University, Lund, Sweden; ^2^ Nuffield Department of Medicine, CAMS Oxford Institute, University of Oxford, Oxford, United Kingdom

**Keywords:** supported lipid bilayer (SLB), lifetime (τ), affinity, ligand receptor binding, fluorescence microscopy, single-molecule imaging and tracking, T cell

## Abstract

Binding between protein molecules on contacting cells is essential in initiating and regulating several key biological processes. In contrast to interactions between molecules in solution, these events are restricted to the two-dimensional (2D) plane of the meeting cell surfaces. However, converting between the more commonly available binding kinetics measured in solution and the so-called 2D binding kinetics has proven a complicated task since for the latter several factors other than the protein-protein interaction per se have an impact. A few important examples of these are: protein density, membrane fluctuations, force on the bond and the use of auxiliary binding molecules. The development of model membranes, and in particular supported lipid bilayers (SLBs), has made it possible to simplify the studied contact to analyze these effects and to measure 2D binding kinetics of individual protein-protein interactions. We will in this review give an overview of, and discuss, how different SLB systems have been used for this and compare different methods to measure binding kinetics in cell-SLB contacts. Typically, the SLB is functionalized with fluorescently labelled ligands whose interaction with the corresponding receptor on a binding cell can be detected. This interaction can either be studied 1) by an accumulation of ligands in the cell-SLB contact, whose magnitude depends on the density of the proteins and binding affinity of the interaction, or 2) by tracking single ligands in the SLB, which upon interaction with a receptor result in a change of motion of the diffusing ligand. The advantages and disadvantages of other methods measuring 2D binding kinetics will also be discussed and compared to the fluorescence-based methods. Although binding kinetic measurements in cell-SLB contacts have provided novel information on how ligands interact with receptors *in vivo* the number of these measurements is still limited. This is influenced by the complexity of the system as well as the required experimental time. Moreover, the outcome can vary significantly between studies, highlighting the necessity for continued development of methods to study 2D binding kinetics with higher precision and ease.

## 1 Introduction

**FIGURE 1:**
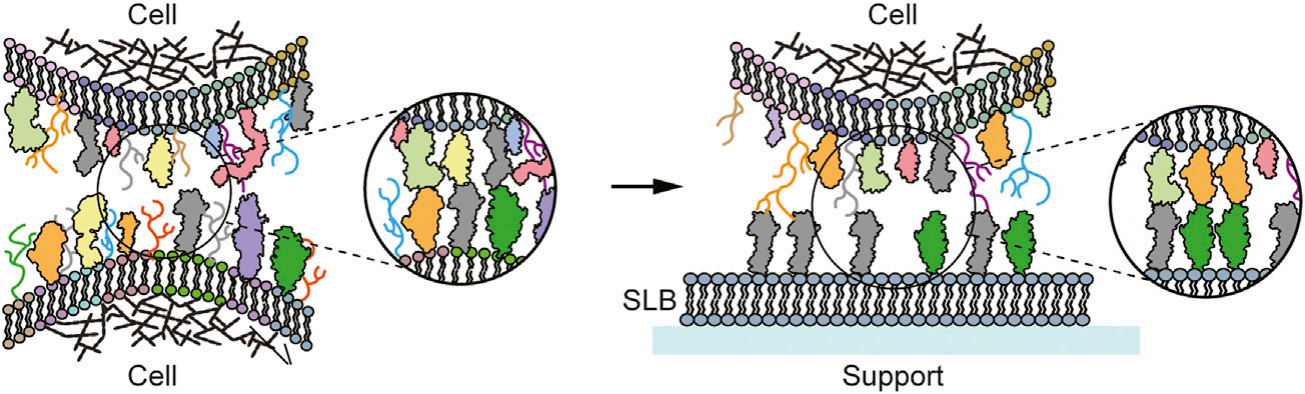
Schematic of a cell-cell vs cell-SLB contact. Left: Cell-cell contact with the cell membrane depicting the different lipids, sugars, and proteins. Trans-interaction between the membrane proteins take place, forming the cell-cell contact. Right: Cell-SLB contact. The SLB replaces one of the contacting cells, but with a simplified composition, here containing only one type of lipid and two types of protein ligands.

The cell membrane forms a barrier between the intracellular and the extracellular environment to preserve the conditions necessary for cellular processes. While acting as a barrier, the cell membrane also allows for transport and signal transduction. To accomplish this the membrane is composed of a lipid bilayer containing a large variety of membrane proteins that can interact with the surrounding environment. These membrane proteins can diffuse laterally in the fluid membrane and are crucial for vital tasks such as cell-cell recognition, signal transduction, endocytosis and ion transport (Céspedes et al., 2020). In addition, binding between protein ligands and receptors across cell-cell contacts are governing a wide range of events from cell signaling to cell mobility and adhesion (Bierer and Burakoff, 1988; Springer, 1990; Dustin, 2019; Trebak and Kinet, 2019). To better understand these processes binding kinetic data are of great value. However, measurements of these so-called two-dimensional (2D) binding kinetics are hampered by the complexity of the cell membrane, and experimental data on 2D binding kinetics have been limited. Therefore, a more simplistic system is needed where one or both of the contacting cell surfaces are replaced with a model membrane allowing for controlled composition of lipids and proteins (Chan and Boxer, 2007; Loose and Schwille, 2009; Zhao et al., 2014). A model system, that was used already in the 1980s to study cell-cell interactions, is the supported lipid bilayer (SLB) (McConnell et al., 1986). The SLB consists of a lipid bilayer resting on a solid surface that can be functionalized with molecules such as protein ligands (Figure 1). Different methods of functionalizing the SLB exist, and we will discuss some of the most common ones in this review.

Experimentally, measuring 2D binding kinetics is more challenging than measuring the corresponding interaction between proteins in bulk solution, henceforth termed three-dimensional (3D) binding kinetics. Several attempts have been made to theoretically connect 2D and 3D binding kinetics, but despite progress in this area, it remains a complicated task since several other factors than the protein-protein interaction per se can have a significant impact on binding in 2D. Such factors are for example protein density (Reister-Gottfried et al., 2008; Junghans et al., 2020) and auxiliary binding molecules (Jönsson et al., 2016; Junghans et al., 2020). Similarly, applied force on the bond (Depoil and Dustin, 2014) as well as membrane fluctuations (Weikl et al., 2019) can have an important influence on the binding kinetics, although this can differ significantly depending on the system used to study this. These discrepancies stress the need for having a controlled system when measuring 2D binding kinetics and we will discuss these effects in more detail in this review.

Different methods of measuring 2D binding kinetics using fluorescence microscopy have emerged to study ligand-receptor interactions in a cell-SLB contact. Brian and McConnell studied already in the mid-1980s the interaction between functionalized SLBs and cytotoxic T cells (Brian and McConnell, 1984) and some years later McCloskey and Poo used fluorescence microscopy to measure the accumulation of fluorescent receptors in the contact between a cell and a liposome (McCloskey and Poo, 1986). In the mid-1990s, Dustin et al. (1996) were the first to measure 2D binding affinity in a cell-SLB contact between the adhesion molecules CD2 and CD58, measurements that were refined in the following year (Dustin et al., 1997). Later, different groups also used cell-SLB contacts and fluorescence microscopy to image the distribution of different molecules in the immunological synapse (Dustin et al., 1998; Grakoui et al., 1999). Altogether, these studies paved the way for using fluorescence-based assays to determine the binding affinity of a receptor-ligand complex, which is achieved by measuring the accumulation of ligands in the cell-SLB contact at different ligand densities on the SLB (Zhu et al., 2007; Dustin, 2009). However, these studies were limited by the necessity to take measurements at steady-state and thus only obtained the affinity of the binding pair and not the rate constants or lifetimes. Later, different single-molecule imaging methods were developed from which one could get the 2D lifetime of different interactions in addition to the affinity on a single bond level (Huppa et al., 2010; O’Donoghue et al., 2013). We will here summarize some of the main findings observed with these methods and discuss various advantages and disadvantages of the different methods measuring 2D binding kinetics as well as recent developments in this area.

Other methods of measuring 2D binding affinity also exist and are based on measuring the likelihood and lifetime of individual bonds forming when a cell and a ligand-coated surface are brought into contact mechanically (Zhu et al., 2013). In addition to estimating an affinity and measuring lifetimes, mechanical-based methods can also be used to study how force influences the bond lifetime, which depending on the interaction can have a significant impact (Depoil and Dustin, 2014). We will at the end of this review discuss different mechanical-based methods and how the results from these studies aid the findings from the fluorescence-based methods mentioned above. Taken together, 2D binding kinetics can be measured with various methods that make use of different model membrane systems to isolate the studied interaction. Each method has different advantages and by using a combination of these approaches we can get a more comprehensive understanding of binding kinetics in 2D and how they compare to their 3D counterparts. Despite the constant development and increase in accuracy of these techniques, there are still many ambiguous results in this area. Moreover, measuring weak interactions between ligands and receptors is still, in some instances, a challenging task that would greatly benefit from improved accuracy and the development of more robust methods.

## 2 General 2D and 3D Adhesion Theory

Studying the binding kinetics of a protein-protein interaction entails the detection of the binding affinity and the lifetime of the studied receptor-ligand complex. The lifetime, *τ*_off_, of a protein-protein complex describes the average time between the formation and dissociation of the complex*,* whereas the dissociation constant *K*_d_ (1/affinity) is the concentration of ligands where half of the receptors have bound a ligand:

$$K_{\text{d}}=\;\frac{\left[\text{R}\right]\left[\text{L}\right]}{\left[\text{RL}\right]}\;$$ (1)

where [R] is the concentration of free receptors, [L] the concentration of free ligands and [RL] the concentration of formed receptor-ligand complexes. For 3D binding events occurring between a sensor surface immobilized with receptors at a concentration [R]_max_ and freely diffusing ligands in solution, Eq. 1 becomes:

$$\left[\text{RL}\right]=\frac{{\left[\text{R}\right]}_{\text{max}}\left[\text{L}\right]}{\left[\text{L}\right]+K_{\text{d}}^{3\text{D}}}\;$$ (2)

For protein binding in a cell contact the situation is slightly different. First, whereas the $K_{\text{d}}^{3\text{D}}$ is expressed in units of volume, the $K_{\text{d}}^{2\text{D}}\;$ is expressed in molecules per area. Similarly, while [R], [L], and [RL] in 3D are expressed in units of molar concentration, the 2D counterparts are given in units of molecules per area. Second, for 2D binding, [R]_max_ is no longer a constant as both ligands as well as receptors are mobile and can diffuse into the cell contact. Hence, Eq. 2 changes to:

$$\left[\text{RL}\right]=\frac{N_{\text{tot}}\times f}{S_{\text{cell}}}\times \frac{\left[\text{L}\right]}{p\left[\text{L}\right]+K_{\text{d}}^{2\text{D}}}\;$$ (3)

where *N*_tot_ × *f*/*S*_cell_ is the density of mobile receptors on the cell surface, *f* is the fraction of mobile receptors, and *p* is the ratio between the contact area and the total cell surface area, *S*_cell_ (Zhu et al., 2007). The maximum density of bound complexes is now given by *N*_tot_ × *f*/(*S*_cell_ × *p*) and is a factor of *f*/*p* larger than the initial receptor density on the cell. For practical reasons the expression in Eq. 3 is commonly written as:

$$\frac{B}{F}=\frac{N_{\text{tot}}\times f}{S_{\text{cell}}\times K_{\text{d}}^{2\text{D}}}-\frac{B\times p}{K_{\text{d}}^{2\text{D}}}\;$$ (4)

where [RL] and [L] are designated as *B* and *F*, respectively. This expression is known as the Zhu-Golan expression and predicts a linear relation between the relative accumulation of ligands, *B*/*F*, in the contact and the parameter *B* × *p* (Zhu et al., 2007). The slope is given by $-1/K_{\text{d}}^{2\text{D}}$ and the *x*-intersect is equal to the density of the total amount of mobile receptors on the cell surface, *N*_tot_ × *f*/*S*_cell_.

The *K*_d_ in 2D is obtained by the slope in Eq. 4 and connects to its 3D counterpart via a characteristic confinement length, *h* (Bell, 1978; Bell et al., 1984):

$$K_{\text{d}}^{2\text{D}}=h\times K_{\text{d}}^{3\text{D}}$$ (5)

In theory, this confinement length can be motivated by the ligands and receptors forming an encounter complex that in turn can react to form the bound state (Bell, 1978). Under the assumption that the forward and reverse rates from the encounter complex to the bound state are the same in 2D and 3D, then *h* approximately corresponds to the maximum distance between a ligand and a receptor entering the encounter complex (Bell, 1978). Although this assumption can be influenced by several parameters such as the flexibility of the molecules (Pierres et al., 1998; Wu et al., 2011) typical values of *h* obtained by comparing 2D and 3D data for the same interaction are of the order of 1 nm, the same value as was theoretically estimated by Bell in 1984 (Bell et al., 1984).

Under the same assumptions, the expressions given by Bell (Bell, 1978) can be used to derive an expression for the lifetime of the interaction in 2D and 3D yielding:

$$\tau_{\text{off}}^{2\text{D}}=\tau_{\text{off}}^{3\text{D}}\times \left(1+\frac{1}{2\pi \left(D_{\text{m}}\left(\text{L}\right)+D_{\text{m}}\left(\text{R}\right)\right)\times \tau_{\text{off}}^{3\text{D}}\times K_{\text{d}}^{2\text{D}}}\right)$$ (6)

where $\tau_{\text{off}}^{2\text{D}}$ and $\tau_{\text{off}}^{3\text{D}}$ are the 2D and 3D lifetime of the interaction, respectively, *D*_m_ (L) is the diffusivity of the ligands and *D*_m_ (R) the diffusivity of the receptors. From Eq. 6 it can be observed that, under these conditions, the 2D lifetime is always larger, or in the limit equal to, the 3D lifetime. In practice, for a ligand-receptor interaction such as a T-cell receptor (TCR) binding its cognate peptide major histocompatibility complex (pMHC) molecule we can estimate: $\tau_{\text{off}}^{3\text{D}}$ = 10 s, $K_{\text{d}}^{2\text{D}}$ = 10 molecules/µm^2^ and *D*_m_ (L) = *D*_m_ (R) = 0.05 μm^2^/s, which inserted into Eq. 6 gives $\tau_{\text{off}}^{2\text{D}}$ = $1.02\tau_{\text{off}}^{3\text{D}}$ . Thus, the 2D and 3D lifetime would for this case be similar, an observation that appears to be valid at least for some experimental systems studied (O’Donoghue et al., 2013; Hong et al., 2015).

## 3 Supported Lipid Bilayers for 2D Binding Studies

**FIGURE 2:**
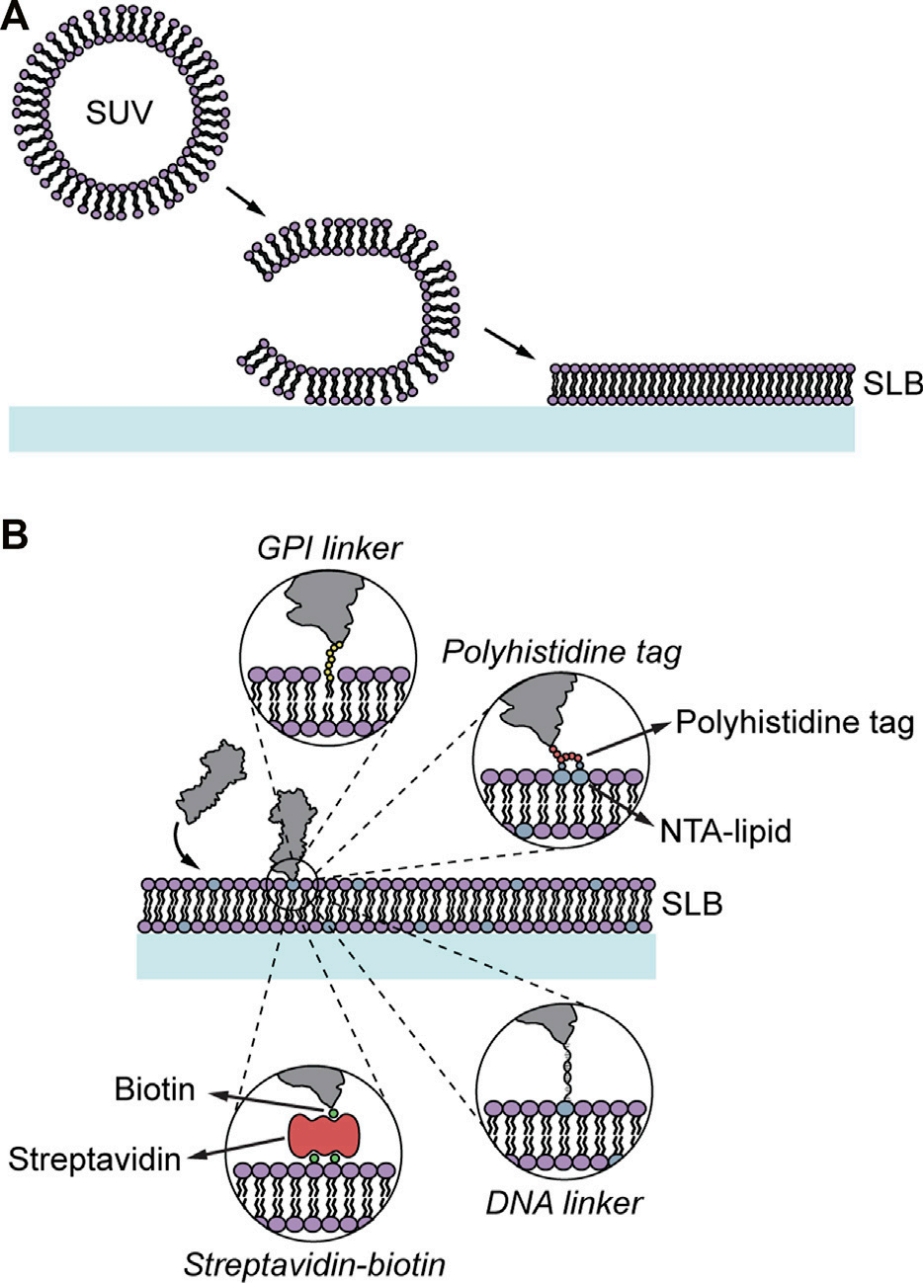
Formation and modification of SLBs. **(A)** SLB formation through vesicle adsorption and rupture. The vesicles adsorb onto a clean glass support, where they rupture and unfold forming an SLB. **(B)** SLBs can be functionalized with proteins using different anchor strategies including GPI linkers, nickel-chelating lipids binding polyhistine-tagged proteins, biotinylated lipids binding, *via* streptavidin, biotinylated proteins, and DNA linkers.

One of the most used membrane mimics is the SLB, which is a lipid bilayer supported by a substrate, typically a flat cover glass slide. The composition of this model membrane can be chosen with high accuracy and is compatible with different surface-based techniques, including fluorescence microscopy (Lichtman and Conchello, 2005; Céspedes et al., 2020). Furthermore, the SLB can be functionalized with proteins resulting in a simplified, flat, and stable cell membrane mimic which facilitates protein-interaction studies. There are various methods to produce SLBs including the Langmuir-Blodgett monolayer transfer or vesicle adsorption and rupture method (Castellana and Cremer, 2006), where the latter is arguably the most used method today. In this method, small unilamellar vesicles of a desired lipid composition adsorb from solution onto the substrate upon which they deform and rupture into a continuous fluidic lipid bilayer (Figure 2A). However, it should be noted that while this method is straightforward for simpler lipid compositions, forming SLBs with a more complex composition involving e.g., charged lipids, cholesterol and phase separating lipids, can be challenging and in many cases not possible (Hardy et al., 2013). In addition, cell membranes show a high level of asymmetry in lipid composition between inner and outer leaflets (Lorent et al., 2020). This asymmetry can to some degree be mimicked in SLBs when using Langmuir-Blodget monolayer transfer, but is more complicated when using vesicle adsorption and rupture to form the SLB (Céspedes et al., 2020). Studies of 2D binding kinetics typically have been restricted to SLBs with phosphatidylcholine as the main membrane component. Information about the influence more complex lipid mixtures have on the binding between ligands and receptors are largely lacking. To be able to investigate this, full-length transmembrane proteins should be incorporated into the model membrane, something that is generally challenging when working with SLBs.

The distance between the SLB and the underlying solid supporting substrate is of the order of 1 nm, which results in proteins spanning the SLB will be hindered by the underlying substrate and not be mobile (Tanaka and Sackmann, 2005). Different approaches using polymer-supported lipid bilayers have been developed to increase the distance between the support and the lipid bilayer to circumvent this, allowing for incorporation of mobile, transmembrane proteins (Tanaka and Sackmann, 2005; Pace et al., 2015). However, incorporating a functional transmembrane protein into a lipid bilayer is by itself challenging and requires careful choice of insertion methods and solubilizers used (Jørgensen et al., 2017). Therefore, in the majority of 2D binding kinetic studies, only the extracellular part of the studied protein has been used. This simplifies the SLB preparation considerably but comes with the drawback that the influence the membrane composition can have on the binding kinetics is omitted. There are several approaches to anchor the extracellular domains of a protein to SLBs (Figure 2B): 1) Proteins can be modified with a glycosylphosphatidylinositol (GPI) linker at the c-terminus that intercalates into the bilayer while forming the SUVs (Paulick and Bertozzi, 2008). 2) Polyhistidine-tagged proteins can bind non-covalently to so-called nickel-chelating lipids in the bilayer (Nye and Groves, 2008). These lipids have a nitrilotriacetic acid (NTA) modified head group that forms coordination compounds with nickel-ions to which histidine can bind (Dietrich et al., 1995). 3) Lipid head groups can also be biotinylated, which allows the binding of a biotinylated protein via a streptavidin linker to the lipid (Liu et al., 1995). Whereas the GPI-linker and the biotin-streptavidin interaction are strong the polyhistidine tag binding to the nickel-chelating lipid is weaker and the bound proteins can detach during an experiment (Nye and Groves, 2008). To minimize this effect polyhistidine-tags with more histidine repeats (>10xH) are often used. Prolonging incubating times of the polyhistidine-tagged proteins with the lipid bilayer has also been shown to have a stabilizing effect (Nye and Groves, 2008). Disadvantages of using GPI-linked proteins are that the GPI linker itself can induce protein aggregation (Dustin and Groves, 2012) and that the preparation of GPI anchored proteins is, in general, more complex compared to the production of polyhistidine-tagged proteins. A problem of biotin-streptavidin linked proteins is the addition of an, at least, extra 5 nm to the overall protein size, which can be particularly problematic in cell-SLB interface studies where the height of the molecules vs the cell-SLB gap is important. 4) The SLBs can also be functionalized with DNA grafted onto the lipids, to which an opposite DNA strand, containing the studied protein ligand, is added (Wilhelm et al., 2021). This allows for a versatile tool, particularly by being able to alter the number of nucleotides making it possible to change the length of the DNA tether as well as the interaction strength between two DNA strands. The latter has for example been used to vary the binding strength of ligands in a chimeric antigen receptor system (Taylor et al., 2017). It is also possible to use DNA origami to control the lateral position of two or more ligands relative to each other in a cell-SLB contact (Hellmeier et al., 2021). For all these methods, care must be taken such that the cells do not bind non-specifically to the model membranes. This has been shown to be particularly important when using nickel-chelating lipids, which can bind to cell receptors on the approaching cell and potentially cause cell signaling (Dam et al., 2021). However, by using different approaches to passivate the SLB, such as adding bovine serum albumin or polyethylene glycosylated lipids in the SLB, the unspecific binding events can be minimized (Taylor et al., 2017; Dam et al., 2021). It should be stressed that the sample preparation is the first crucial step in 2D binding kinetic studies using SLBs and thus verifying the properties of the deposited SLB should be done before performing any interaction experiment. This includes checking for unspecific binding as discussed above, and to measure the lipid and protein mobility using, e.g., fluorescence recovery after photobleaching (FRAP) to measure the mobility of lipids and proteins in the SLB (Lorén et al., 2015).

## 4 2D Binding Kinetics Measured Using the Zhu-Golan Method

**FIGURE 3:**
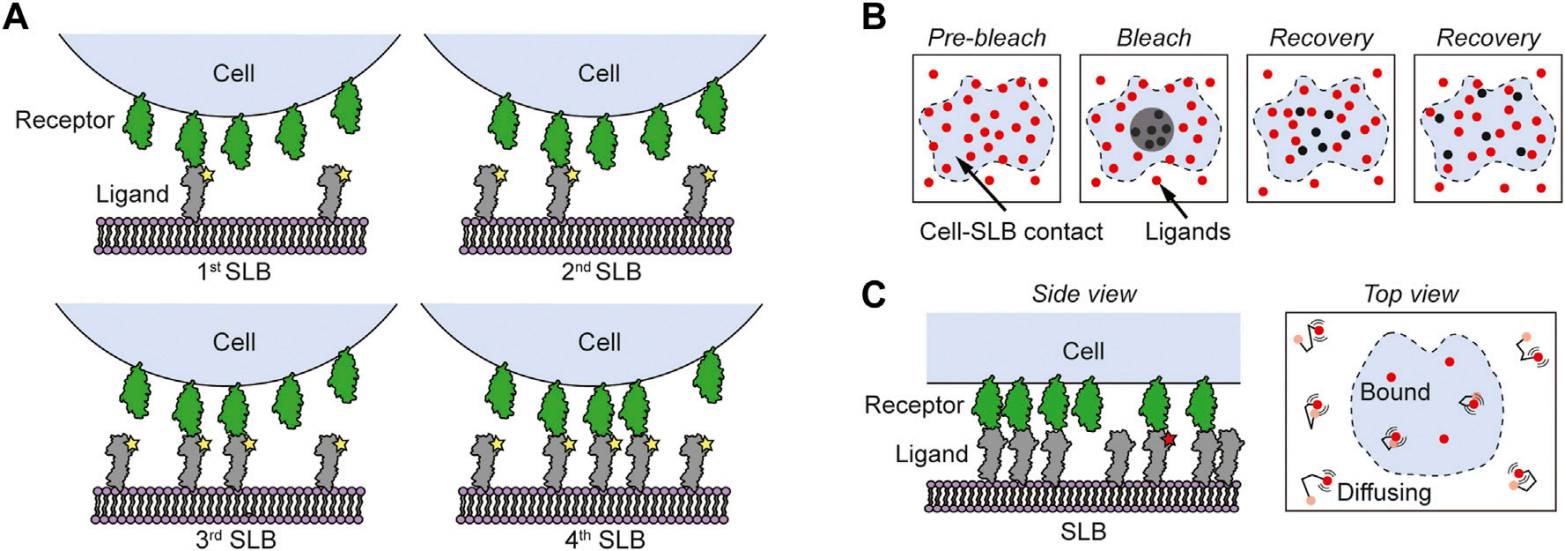
Different fluorescence-based methods for measuring 2D binding kinetics in cell-SLB contacts. **(A)** In the Zhu-Golan method cells expressing the studied receptor interact with SLBs carrying the corresponding ligand. By measuring the accumulation of fluorescently labeled ligands in cell-SLB contacts for SLBs with different densities of ligands, the 2D *K*_d_ of the interaction can be obtained using Eq. 4. **(B)** Using FRAP a delimited area of the ligands (red circles) in the cell-SLB contact (blue area) is photobleached and by following the rate of recovery the lifetime of the interaction can be estimated. **(C)** Single-molecule fluorescence microscopy is used to track the motion of fluorescently labeled ligands (red circles) in the cell-SLB contact (blue area). Unlabeled ligands are here included to facilitate the formation of the cell-SLB contact. When the ligands bind, they stop moving, and from this the average 2D lifetime of the interaction can be determined.

Early fluorescence-based 2D binding studies especially revolved around the CD2-CD58 interaction between T cells and antigen presenting cells. In 1996, Dustin and colleagues obtained a binding affinity of 21 molecules/μm^2^ for this interaction by studying CD58-functionalized SLBs interacting with CD2-presenting Jurkat T cells (Dustin et al., 1996) using an analysis method proposed by Scatchard (1949) in the late 1940s. However, the Scatchard method considers the receptor concentration to be constant, an assumption that is not valid in a cell-SLB contact. To correct for this, an improved analysis model to determine the 2D binding affinity, the so-called Zhu-Golan model (Eq. 4), was developed (Dustin et al., 1997; Zhu et al., 2007). In these measurements, the accumulation of fluorescently labeled ligands in the cell-SLB contact is measured for SLBs containing different densities of ligands (Figure 3A). By fitting this data to the Zhu-Golan expression in Eq. 4 the 2D *K*_d_ of the receptor-ligand interaction can be obtained. It is important to emphasize here that the cell-SLB area is a highly dynamic environment. The contact area grows over time until it reaches a steady-state value, which is a function of the initial ligand density in the SLB (Shao et al., 2005; Junghans et al., 2020). A period of ∼30 min is typically required for ligand-functionalized bilayers to reach steady state upon interaction with receptor-expressing Jurkat T cells (Dustin et al., 1997; Zhu et al., 2007; Chouliara et al., 2021).

The Zhu-Golan analysis requires the measurement of the concentration of bound and free ligands, as well as the cell-area fraction that is in contact with the SLB. One complicating factor is that, due to steric effects, the density of free ligands in the cell-SLB contact is generally lower compared to the density on the SLB outside the contact (Dustin, 2009). To estimate the free ligand density in the cell-SLB contact a non-binding protein of similar size has often been used as an addition to the ligand of question. By using this approach it has been shown that the density of free ligands in the contact is typically reduced by 25–50% among the cells for proteins of 5–10 nm height (Zhu et al., 2007; Jönsson et al., 2016; Junghans et al., 2020).

The Zhu-Golan method has been used to obtain 2D *K*_d_s for a range of protein-protein interactions. For example, the 2D *K*_d_ of the human CD2-CD58 interaction has been found to be ∼6 molecules/μm^2^ (Zhu et al., 2007), whereas the corresponding rat CD2-CD48 interaction had an almost order of magnitude higher 2D *K*_d_ of ∼40 molecules/μm^2^ (Dustin et al., 1997; Jönsson et al., 2016). Compared to the corresponding 3D *K*_d_ values of 10 and 60 µM (Davis et al., 2003), for human CD2 binding CD58 and rat CD2 binding CD48, respectively, this gives, using Eq. 5, a similar *h* value of around 1 nm for both interactions. In addition, the interaction between rat CD2 binding different mutants of CD48 has also been studied. For rat CD48_T92A_, a high affinity mutant of CD48, a 2D *K*_d_ of 6 molecules/μm^2^ was obtained by Junghans et al. (2020), and Jönsson et al. (2016) measured the 2D *K*_d_ for rat CD48_Q40R_, a weaker binding mutant of CD48, to be 380 molecules/µm^2^. With a 3D *K*_d_ of 11 µM for the rat CD48_T92A_ interaction (Evans et al., 2006) and a 3D *K*_d_ of 440 µM for the rat CD48_Q40R_ interaction (Jönsson et al., 2016) this results in *h* values of 0.9 and 1.4 nm for rat CD2 binding to rat CD48_T92A_ and rat CD48_Q40R_, respectively. Furthermore, two other studies used the Zhu-Golan method to measure binding kinetics of the CD28-CD80 interaction, which provide a costimulatory signal in T-cell activation, obtaining a 2D *K*_d_ of 1 molecule/μm^2^ (Bromley et al., 2001), and of L3-12 TCR binding to the pMHC class II molecule HLA-DQ8 containing the glia-α1-peptide, of 14 molecules/μm^2^ (Junghans et al., 2020). Comparing with the 3D *K*_d_ values of 2 and 7 µM for CD28-CD80 (Bromley et al., 2001) and L3-12 TCR binding HLA-DQ8-glia-α1 (Broughton et al., 2012), this gives an *h* of 0.8 nm for CD28-CD80 and an *h* of 3 nm for L3-12 TCR binding HLA-DQ8-glia-α1. Why the TCR-pMHC interaction has a larger *h* value than the other interactions mentioned here, and thus also a relatively weaker 2D vs 3D affinity, is not known, but it highlights the possible differences when comparing proteins restricted to a membrane and proteins free in solution. The Zhu-Golan method is also well suited to study weak interactions. For example, Jönsson et al. (2016) used this to study the CD4-pMHC class II interaction obtaining a 2D *K*_d_ of 5000 molecules/μm^2^, as far as we know the lowest 2D affinity reported to date for a receptor-ligand interaction (Jönsson et al., 2016). A corresponding 3D *K*_d_ value for this interaction has so far been too weak to measure.

Obtaining accurate values for binding affinities using the Zhu-Golan method requires measuring over several cell-SLB contacts for each ligand concentration. This is due to an inherent spread in accumulation for each cell contact caused by the spread in receptor density in the cell population (Jönsson et al., 2016; Chouliara et al., 2021). It has further been shown that cells expressing a higher number of surface receptors have a higher probability to bind to SLBs with low ligand densities, which can influence the outcome of the Zhu-Golan analysis (Dustin et al., 1997). A possible route to circumvent this is to regulate the density of ligands in the SLB after the cells have made contact. This can for example be done by adding more polyhistidine-tagged ligands to the solution, exploiting the fact that the histidine-NTA interactions are typically orders of magnitude stronger than the interaction between the ligand and the receptor, thus minimizing any undesirable binding to the cell surface outside of the cell-SLB contact. Junghans et al. (2020) used this approach to determine 2D *K*_d_ values of different ligand-receptor pairs with values similar to the ones obtained by the classical Zhu-Golan approach. However, in this approach the *K*_d_ obtained from single cell-SLB contacts was found to vary quite significantly (Junghans et al., 2020; Chouliara et al., 2021), making this approach less suitable to determine single-cell *K*_d_ values. For this purpose, it instead appears advantageous to start at a high density of ligands in the SLB and reduce the density of polyhistidine-tagged ligands in steps using, for example, imidazole as done by Chouliara et al. (2021). This was used to obtain accurate single-cell affinities of rat CD2 binding rat CD48_T92A_ expressed on Jurkat T cells, showing only a minor spread in 2D *K*_d_ values over the whole cell population.

## 5 Single-Molecule Methods to Measure 2D Binding Kinetics

Whereas the Zhu-Golan method has been used extensively to study 2D affinities of various protein-protein pairs, it does not give information on the 2D lifetime of the interaction. For this purpose, FRAP has instead been used. In FRAP, a delimited area of fluorescent molecules in the cell-SLB contact is photobleached and the recovery of the intensity due to diffusion and binding of new fluorescent ligands is recorded (Figure 3B) (Dushek et al., 2008; Tolentino et al., 2008; Wu et al., 2008). Using this approach Tolentino measured a 2D lifetime of 14 s for human CD2 binding CD58, which is two orders of magnitude longer than the corresponding 3D lifetime (Tolentino et al., 2008). However, fitting the recovery over time in the cell-SLB contact is in general non-trivial and for short lifetimes the recovery will be dominated by diffusion. This gives a limit of 5–10 s for how short lifetimes that could still be accurately measured when using a 5–10 µm wide bleach spot (Jönsson et al., 2016; Junghans et al., 2020). However, since this limit scales with the initial width of the bleach spot squared it can in theory be shortened substantially (Jönsson et al., 2016).

In recent years, a method that has become dominant to measure short lifetimes is single-molecule imaging and tracking. The position of a single fluorescently labeled molecule can be determined with nm spatial resolution allowing for single-particle tracking of the molecule until it is bleached (Manzo and Garcia-Parajo, 2015) (Figure 3C). Single-particle tracking also makes it possible to detect protein binding events or protein association by distinguishing bound and free proteins either from a change in motion in the molecule’s trajectory (Axmann et al., 2012) or from reduced motion blur when a protein is bound (O’Donoghue et al., 2013). To further distinguish between bound and free ligands both the receptors and the ligands can be labelled with different fluorophores that act as a fluorescence resonance energy transfer (FRET) pair. Here a FRET signal is shown only when the ligand and receptor are bound, which was used by Huppa et al. (2010) to measure the 2D binding kinetics of 5c.c7 TCR and 2B4 TCR binding to different pMHC molecules (IE^k^/MCC, IE^k^/T102S and IE^k^/K99R). By comparing the median 2D *K*_d_ value in a cell-SLB contact with the corresponding 3D value *h* values ranging between 1.2 and 2.5 nm were obtained. The smallest *h* value was found for the weakest interaction, whereas the higher *h* values were found for pMHC molecules with highest (agonist) potency for T-cell activation. Interestingly, the interaction between the also agonistic L3-12 TCR binding HLA-DQ8-glia-α1 gave an *h* of 3 nm (Junghans et al., 2020), a factor of three higher than the “typical” 1 nm obtained from many other ligand-receptor interactions. Whether this is a coincidence or agonistic TCR-pMHC interactions, in general, have a higher *h* value can at the moment only be speculated.

Interestingly, the 2D affinities measured by Huppa et al. were of similar magnitude at 24 and 37°C (Huppa et al., 2010), a correlation that has also been observed by us for CD4 binding pMHC class II molecules (Jönsson et al., 2016) and for rat CD2 binding rat CD48_T92A_ (Junghans et al., 2020; Dam et al., 2021). Nonetheless, Huppa et al. (2010) observed a monotonic decrease from 1.7 to 0.1 s in 2D lifetimes as the temperature was increased from 24 to 37°C. A similar lifetime at 37°C for this interaction was found later by Axmann et al. (2012) using a complementary single-molecule tracking method. Huppa et al. (2010) further found that the obtained 2D lifetimes were 3–12-fold shorter than the corresponding lifetimes measured in 3D, a difference that was reduced upon treating the cells with actin-depolymerizing drugs. This indicates that forces from the cytoskeleton can act on the bond reducing the lifetime of the interaction, a characteristic of a so-called slip bond. In contrast, O’Donoghue et al. (2013) tracked individual complexes of AND TCR and 5c.c7 TCR binding to IE^k^/MCC in live T cell-SLBs contacts and saw no influence on the 2D lifetime with and without actin-depolymerizing drugs, which in addition was similar to the lifetime measured in solution. Furthermore, no difference in the 2D lifetime of 5c.c7 TCR binding to IE^k^/MCC with temperature was observed (O’Donoghue et al., 2013). The reason for the diverse results between these studies is unclear, however, one possible explanation has been that Huppa et al. measured in the periphery of the immunological synapse instead of in the center, indicating that the local environment can influence the binding kinetics (Depoil and Dustin, 2014).

Pielak et al. (2017) used single-molecule imaging to quantify the ratio of bound and free ligands in cell-SLB contacts for T cells with AND TCR and 5c.c7 TCR binding SLBs with different pMHC molecules including IE^k^/MCC (Pielak et al., 2017). By also labelling the TCR in the cell membrane the number of receptors in the cell-SLB contact could be determined and from this the 2D *K*_d_ of the interaction. Overall, it was found that there was an affinity optimum when the density of the pMHC molecules ranged from 1 to 10 molecules/µm^2^. In addition, the 2D affinities obtained by Pielak et al. (2017) were weaker than those measured by Huppa et al. (2010). For example, the 2D *K*_d_ for 5c.c7 TCR binding IE^k^/MCC at 37°C was measured to be 39 molecules/µm^2^ by Huppa et al. (2010), whereas Pielak et al. (2017) had a value of 110–400 molecules/µm^2^ depending on the density of pMHC molecules in the SLB. With a 3D *K*_d_ of 40 µM for this interaction (Huppa et al., 2010), this corresponds to *h* = 1.6 nm for the data from Huppa et al. (2010) and *h* values ranging from 4.6 to 17 nm for the data from Pielak et al. (2017). The latter values are, compared to other measured interactions with fluorescence-based methods, quite high. A possible influencing factor to this might be that not all receptors detected in the cell-SLB contact by Pielak et al. (2017) are capable of binding pMHC molecules in the SLB, due to e.g., membrane undulations (Mege et al., 1986), leading to a too high estimate of the bound receptor density.

Single-molecule imaging has the advantage that rare binding events can be detected since the lifetime distribution instead of the population average is measured. Lin et al. (2019) used this to study binding events between AND TCR expressing T cells and SLBs functionalized with IE^k^/MCC while simultaneously monitoring T-cell activation. Their results showed that T cells responded disproportionately strong to rare, long-dwelling binding events that can be an order of magnitude longer than the average lifetime values. Such findings are not apparent when analyzing and averaging an ensemble of binding events. An additional advantage of using single-molecule imaging is that there is no need to compensate for the exclusion of free ligands in the contact area as compared to in the Zhu-Golan approach. On the other hand, single-molecule imaging experiment are generally more technically demanding than the Zhu-Golan measurements, particularly when it comes to correctly tracking and analyzing the single-molecule data to distinguish between different types of motion.

## 6 The Influence of Auxiliary Molecules on Binding

In a live cell-cell contact there are multiple different interactions acting in unison (Huppa and Davis, 2013). For example, when the TCR binds to pMHC the co-receptors CD4 or CD8 can simultaneously bind to the pMHC molecule influencing the sensitivity of the T cell and potentially also the strength of the TCR-pMHC interaction (Jiang et al., 2011; Glassman et al., 2018). Other molecules, including the adhesion pairs CD2 binding CD58 and lymphocyte function-associated antigen 1 (LFA-1) binding intercellular adhesion molecule 1 (ICAM-1), enable cell-cell adhesion, but how this influences the specific TCR-pMHC binding is not yet fully understood (Huppa and Davis, 2013). Nevertheless, that auxiliary binding molecules can have an important effect on ligand-receptor binding has been shown when measuring the very weak interaction between CD4 and pMHC class II molecules (Jönsson et al., 2016). Crucial to obtaining the 2D *K*_d_ affinity in that study was the use of the auxiliary binding pair human CD2-CD58. The height of the CD2-CD58 pair matches that of CD4-pMHC class II, suggesting that it might help in aligning the two contacting surfaces improving the chances of CD4 binding to the pMHC class II molecule. However, binding of weakly interacting ligand-receptor pairs has also been detected with auxiliary binding proteins that are of substantial different height to the studied interaction pair, which should not allow the two contacting surfaces to align in an optimal way. For example, both Huppa et al. (2010) and Pielak et al. (2017) used the significantly longer binding pair LFA-1 binding ICAM-1 as auxiliary binding molecules and managed to detect weak binding events between the TCR and pMHC molecules.

As stated above, auxiliary molecules can aid in the formation of weaker bonds by simply providing contact adhesion. In general, a minimum ligand density is needed for cell adhesion and contact growth to occur (Dustin et al., 1997; Zhu et al., 2007; Junghans et al., 2020). Where this threshold lies depends on the density of receptors on the contacting cell, as for example SLBs with a low density of ligands selectively bind to cells with the highest density of receptors (Dustin et al., 1997), as well as on the 2D *K*_d_ of the ligand-receptor interaction. Generally, ligand-receptor pairs with a smaller *K*_d_ have a lower density threshold to promote binding. For example, for human CD2 binding CD58 and rat CD2 binding CD48_T92A_, both with a similar 2D *K*_d_ of 6 molecules/µm^2^ (Zhu et al., 2007; Junghans et al., 2020), the density threshold was found to be around 20 molecules/μm^2^ when binding Jurkat T cells containing ∼100,000 receptors in total (Zhu et al., 2007; Junghans et al., 2020), whereas for rat CD2 binding wildtype CD48, which has a 2D *K*_d_ of ∼40 molecules/μm^2^ (Dustin et al., 1997; Jönsson et al., 2016), the threshold density was of the order of 200 molecules/µm^2^ (Dustin et al., 1997). For the weak CD4-pMHC class II interaction, which has a 2D *K*_d_ of 5000 molecules/µm^2^, the threshold density for ligands in the SLB could thus, even for cells with a high number of receptors, be many thousands of molecules/µm^2^, a concentration higher than what is experimentally possible to achieve (Jönsson et al., 2016). Using CD2-CD58 as auxiliary binding molecules could thereby bypass this density threshold necessary for contact formation.

Although auxiliary binding molecules can aid in contact formation and support binding, they can also effectively lower the affinity of other protein-protein interaction pairs. Two examples of this are shown in Junghans et al. (2020) which used rat CD2 binding CD48_T92A_ as the auxiliary binding pair when measuring the affinity of the pMHC class II molecule HLA-DQ8-glia-α1 interacting either with L3-12 TCR or with CD4 (Junghans et al., 2020). The 2D *K*_d_ values for these two interactions are two orders of magnitude different, but for both systems, it was found that when having bound densities of rat CD2-CD48_T92A_ above 300 molecules/µm^2^ the affinity of the studied interaction dropped by a factor of two compared to lower levels of auxiliary binding molecules. In addition, a similar trend was observed when instead treating TCR-pMHC as auxiliary binding molecules and studying the binding affinity of rat CD2 binding CD48_T92A_ (Junghans et al., 2020). At elevated densities of TCR the affinity of the rat CD2-CD48_T92A_ interaction decreased by a factor of 1.5. One possible reason for the lowered affinity could be that there is a mismatch in height between the auxiliary binding molecules and the studied ligand-receptor pair. This could lead to higher exclusion of free ligands in the contact as well as to a higher energy penalty for the bond formation due to the need of deforming the membrane. Auxiliary molecules can thus have multiple roles, at lower densities they can facilitate cell contact and align opposing membranes, whereas at higher densities they can decrease the effective affinity of other interactions in the contact. However, more studies on this effect are needed in order to determine how this influences binding where other auxiliary binding molecules, of different overall length and affinity, are used.

## 7 Membrane Fluctuations and Cooperative Binding

In the previous chapters we discussed different studies that measured binding kinetics in the contact between an SLB and a live cell. Other studies, however, have replaced the cell with a second model system to obtain an even more controlled environment for studying 2D binding kinetics. Here so-called giant unilamellar vesicles (GUVs) have frequently been used. GUVs are vesicles of similar size to that of a live cell (∼10 µm in diameter) and their composition can be controlled during the fabrication process. Contrary to SLBs, GUVs are softer and free-standing allowing for a faster lipid diffusivity in the membrane (Jenkins et al., 2019). Furthermore, they can be functionalized with transmembrane proteins (Dezi et al., 2013), which makes them an attractive model for membrane protein studies. The most common method of GUV production is the rehydration of a lipid film under the influence of an alternating electric field (Angelova and Dimitrov, 1986) although other methods exist as well (Fenz and Sengupta, 2012; Litschel and Schwille, 2021). In addition, GUVs derived from the plasma membrane of cells can also be made (Sezgin et al., 2012; Steinkühler et al., 2018). The GUV-SLB contact has frequently been studied with reflection interference contrast microscopy (RICM) where the distance in the GUV-SLB contact can be measured with nm precision (Limozin and Sengupta, 2009). This allows for membrane fluctuations and subsequent bond regions in the GUV-SLB contact to be studied with both high spatial and temporal resolution (Fenz and Sengupta, 2012). Membrane fluctuations are an inherent part of the elasticity of the cell membrane and get suppressed when the membrane binds to another surface. This in turn results in an energy loss which effectively reduces the affinity of a ligand-receptor interaction, and has been studied extensively using simulations showing, as an example, a quadratic effect on the density of bound ligands with ligand density (Hu et al., 2013; Weikl et al., 2019).

The influence of membrane fluctuations on 2D binding kinetics has also been studied experimentally (Fenz and Sengupta, 2012; Fenz et al., 2017). Two examples of where GUVs have been used to show cooperative binding of ligand-receptor pairs due to membrane fluctuations include binding between cadherin proteins (Fenz et al., 2017) and E-selectin binding Sialyl Lewis X (Reister-Gottfried et al., 2008). In addition, Steinkühler et al. (2018) measured the affinity of CD47 on plasma-derived GUVs binding SIRPα immobilized on a surface, showing an increased cooperativity in binding with ligand density, an effect that was reduced when the membrane was made stiffer (Steinkühler et al., 2018). Interestingly, these effects are not generally observed on cell-SLB systems. For example, Chouliara et al. (2021) showed an almost constant affinity vs the density of bound rat CD2 ligands in the range of 200–600 bound molecules/µm^2^ when binding to rat CD48_T92A_ on Jurkat T cells (Chouliara et al., 2021). It cannot be ruled out that cooperative effects occur at lower ligand densities, but it could be that the interaction with the cytoskeleton makes the cell membrane less sensitive to membrane fluctuations. On the other hand, measurements on latrunculin-treated cells, where the actin cytoskeleton has been disrupted, have, at least for some systems, shown similar binding affinities (Junghans et al., 2020) as well as lifetimes (O’Donoghue et al., 2013) in cell-SLB contacts as compared to untreated cells indicating that other parameters are also involved in regulating membrane fluctuations on live cells and their influence on 2D binding kinetics.

## 8 Mechanical-Based Methods to Study 2D Binding Kinetics

The above presented fluorescence-based methods have the advantage that the fluorescently labeled ligand-receptor complex can be singled out and studied in a contact that contains many other molecules and intercellular bonds. However, the fluorescent labels themselves can influence the measurement and care must be taken to avoid or account for this (Sánchez-Rico et al., 2017). To verify that the fluorescent labels do not interfere with the measurements, controls with different labels and measurements with a mixture of unlabeled and labeled proteins could help to investigate whether the label interferes with the 2D binding kinetics. In addition, methods that do not require fluorescent labelling such as mechanical-based methods, can be used to obtain 2D binding kinetics. These methods do not generally rely on SLBs as a model system for one of the contacting cells but instead use for example ligands immobilized on a surface or ligands anchored to a red blood cell (RBC) as in the micropipette adhesion frequency assay discussed further below. These mechanical-based methods are typically used to measure the initial binding events between ligands and receptors on two contacting cell surfaces distinguishing them even further from fluorescence-based methods.

**FIGURE 4:**
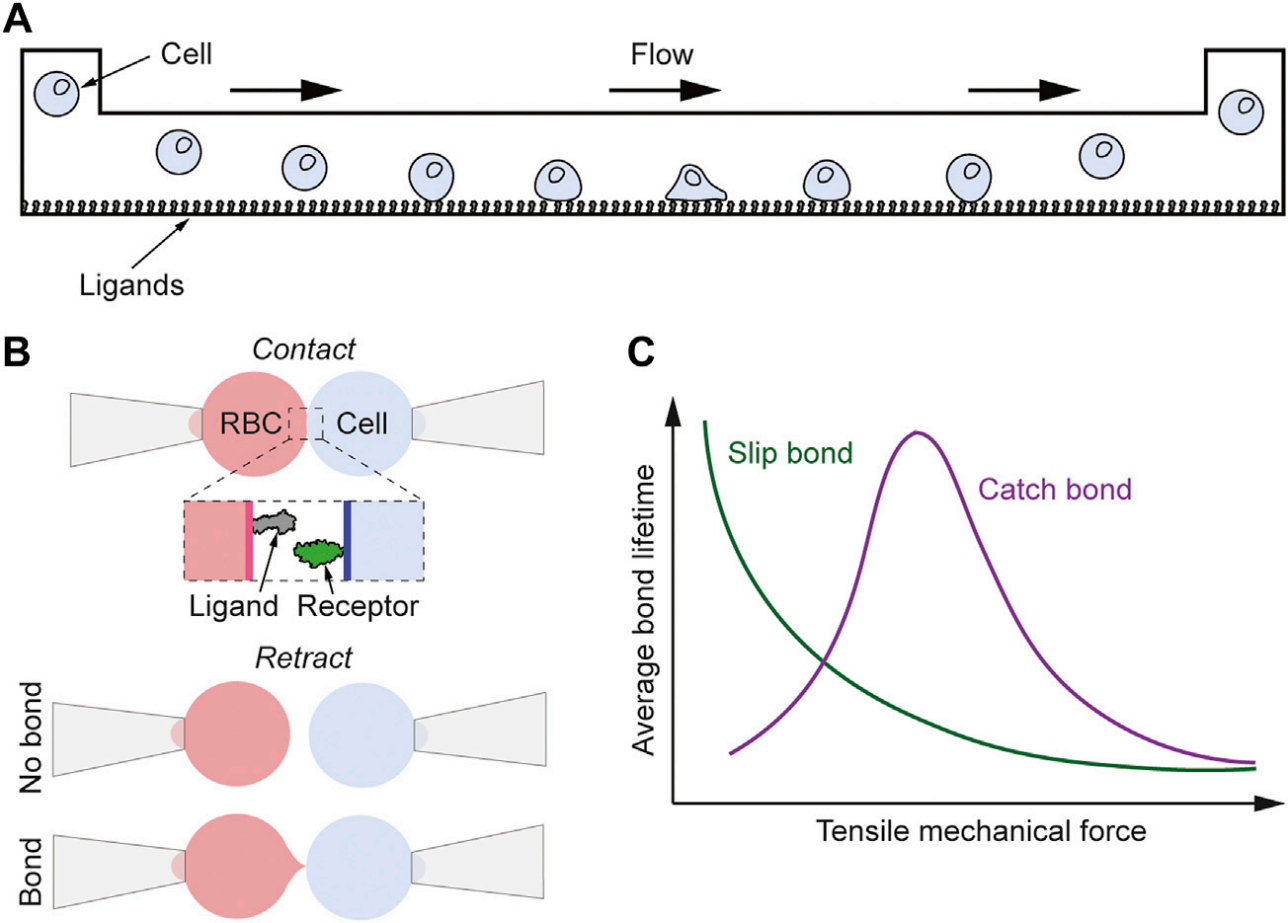
Mechanical-based methods for measuring binding kinetics of ligand-receptor interactions. **(A)** Schematic of the flow chamber method. A cell in the ligand-functionalized flow chamber adheres to the surface for a certain time allowing for the 2D binding kinetics of the interaction to be measured under different flow/force conditions. **(B)** Schematic of the adhesion frequency assay. A bond is detected as a deformation of the RBC when the two cells are retracted from each other, and from the adhesion probability at different contact times the binding kinetic can be determined. **(C)** Schematic graph showing the lifetime vs force behavior for slip and catch bonds.

One of these methods is the flow chamber method, where a microfluidics device with inlet and outlet ports exert a laminar shear flow to receptor-bearing cells moving above a surface (Figure 4A) (Pierres et al., 2008). This surface is coated with ligands at a sufficiently low density in order to ensure single bond formation between the cell and the surface. Successful receptor-ligand interactions lead to the immobilization of the cell for a specific amount of time which is dependent on the proteins’ interaction strength. Continuous microscopic observations of the frequency and the time that the cells adhere on the surface provide information on both the 2D affinity and lifetime of the receptor-ligand complex. This method has for example been used to study the interaction between rat CD2 binding rat CD48 resulting in an off-rate of 7.8 s^−1^ and the bonds showed slip bond behavior when acted upon by force (Pierres et al., 1996). Other examples include measuring the lifetime of P-selectin binding its glycoprotein ligand (Alon et al., 1995) and studies on TCR binding its pMHC ligand (Robert et al., 2012; Limozin et al., 2019). For the latter system, Robert et al. (2012) found similar 2D and 3D lifetimes for the studied 1G4 TCR binding NY-ESO-1 peptide on HLA-A2 (Robert et al., 2012), and Limozin et al. (2019) studied the influence of force on the same TCR-pMHC interaction finding that depending on the peptide some of the interactions showed ideal bond behavior whereas others showed slip bond behavior (Limozin et al., 2019). None of the bonds behaved as catch bonds, independent of T-cell activation potency (Limozin et al., 2019).

Another method to study single bond formation is the adhesion frequency assay in which the interaction between two proteins is detected by using a ligand-coated RBC and a receptor-expressing cell or vesicle (Evans et al., 1991; Evans et al., 1995). Both cells are aspirated onto micropipettes, whereby the RBC is, through micromanipulation, brought in and out of contact with the receptor-expressing cell (Figure 4B). An interaction between the two cells can be detected by the deformation of the RBC occurring as the cell is being pulled away. By repeating this procedure hundreds of times both the 2D lifetime and the effective binding affinity can be obtained (Chesla et al., 1998). Instead of expressing the ligands on the surface of an RBC, which itself can interact with the receptor-carrying cell (Dustin, 2009), the ligands can also be coated onto a glass bead that is attached to the RBC. This approach has been termed the thermal fluctuation assay (Chen et al., 2008). It has the advantage of reducing potential RBC-cell interactions as well as of improving measurement quality and robustness. Both these micropipette-based methods have provided invaluable information on the interaction between different ligands and receptors in 2D. For example, Huang et al. (2010) used this to find that the 2D lifetime of different TCR-pMHC interactions can be orders of magnitude larger than their corresponding 3D values, and the former correlated better with T-cell activation (Huang et al., 2010). In contrast, the same group later obtained similar 2D and 3D lifetimes for other TCR-pMHC interactions (Hong et al., 2015), again indicating that the behavior in 2D binding between different, although in many aspects similar, systems can show significantly different trends. The micropipette-based methods have also been used to study the trimeric TCR-pMHC class I-CD8 interaction showing a cooperative effect in binding between CD8 and TCR (Jiang et al., 2011).

Both the flow chamber method and the micropipette-based methods described above have the advantage, compared to fluorescence-based methods, that they can be used to measure the influence force has on the binding kinetics and are ideal to study initial bond formation. One drawback is that auxiliary binding molecules cannot be used since only individual bonds are measured and thus the influence they have on the binding kinetics cannot be studied. Another issue with the micropipette-based methods is to obtain accurate 2D *K*_d_ values. The micropipette-based methods give effective 2D affinities, defined as $K_{\text{d}}^{2\text{D}}/A_{\text{c}}$ , where *A*_c_ is the contact area (Huang et al., 2010). However, the exact value of the contact area is not known and thus these methods are best used for comparing effective affinities between interactions measured with the same setup. It should also be mentioned that when *A*_c_ is estimated and used to determine the actual 2D *K*_d_ in the micropipette-based method the values obtained are typically orders of magnitude lower than what is observed using fluorescence-based methods indicating differences in binding between newly formed and stable contacts (Dustin et al., 2001; Zhu et al., 2013).

In addition, 2D lifetimes and their dependence on force have been studied by other methods as well, including atomic force microscopy (AFM) and optical tweezers. Both these techniques have in terms of sample preparation as well as data analysis many similarities with the micropipette-based methods described above. For AFM, a cantilever tip is functionalized with a protein ligand which is then repeatedly brought into contact with receptors on an underlying surface, for example an SLB or a cell, to allow for bond formation to be studied (Chang et al., 2012). Retracting the cantilever leads to a continuous increase in the force applied to the formed bond, which results in a jump of the cantilever upon bond rupture. From this data the lifetime of an interaction can be determined (Lee et al., 2007). For example, AFM was used by Zhang et al. (2002) to study the interaction between low and high affinity LFA-1 and ICAM-1 under force (Zhang et al., 2002). Here, an LFA-1 expressing T cell hybridoma was attached to the AFM cantilever and was brought into contact with ICAM-1 coated on a solid support. A fast and slow loading regime of the interaction was identified and 2D kinetic off-rates of 4 s^−1^ and 0.17 s^−1^ for the low and high affinity LFA-1-ICAM-1 interactions, respectively, were obtained. These values were of similar magnitude to the corresponding 3D binding off-rates (Zhang et al., 2002). In another study, Kokkoli et al. (2004) measured the binding and unbinding of α_5_β_1_ integrins to ligands on an SLB using AFM (Kokkoli et al., 2004). The AFM tip was functionalized with the integrins and brought into contact with the ligands on the SLB. This made it possible to obtain single-molecule force spectroscopy data of the interaction resulting in an off-rate of 0.015 s^−1^ in the absence of force, which is similar to the value obtained from measurements in solution (Kokkoli et al., 2004).

In optical tweezers a focused light beam is used to micro-manipulate μm-sized beads coated with ligands (Ashkin, 1992). These beads can be brought into contact with a cell or a model surface containing the corresponding receptor without being in physical contact with the sample. Rinko et al. (2004) used optical tweezers to bring a P-selectin glycoprotein ligand-1 coated polystyrene bead into contact with a selectin-coated glass surface (Rinko et al., 2004). It was found that the rupture force increased with the loading rate and that the 2D off-rate at zero force was 1.4 s^−1^, the same value as measured in solution (Rinko et al., 2004). Rinko et al. (2004) also estimated the 2D *K*_d_ of the interaction to be 0.8 molecules/µm^2^. With a 3D *K*_d_ of 0.3 µM (Rinko et al., 2004), this corresponds to an *h* value of 4 nm, which is of the same magnitude as the values obtained using fluorescence-based methods. Optical tweezers have also been used to measure the lifetime of the TCR-pMHC interaction under force (Das et al., 2015). In this study Das et al. (2015) used pMHC class I molecules coupled to an optically trapped bead and brought the construct into contact with agonist TCRs either attached to a solid surface or expressed on the surface of a T cell. For both systems a catch bond behavior, peaking around 15 pN, was observed. However, the lifetime obtained from the interaction measurements between the pMHC molecules and TCRs on the cell surface were higher than those obtained from the cell-free measurements, a difference that in part could be attributed to simultaneous CD8 binding.

## 9 Force and 2D Binding

It was mentioned in the previous chapter that the mechanical-based methods have a distinct advantage compared to the fluorescence-based methods when it comes to investigating how the interactions depend on an applied force. Theoretically, the influence force has on the bond lifetime was discussed already in the late 1970s by Bell (1978). Bell derived an expression where the bond lifetime decreases exponentially with the application of force, a behavior that later has been termed a slip bond. Many bonds show this behavior in biology (Rakshit et al., 2012). However, there also exist bonds that under low to intermediate force have a longer average lifetime compared to when no force is applied (Figure 4C). These bonds are called catch bonds and the applied force that results in the longest lifetime is typically of the order of 10–30 pN (Rakshit et al., 2012; Liu et al., 2014; Sibener et al., 2018). Examples of catch bonds are P-selectin binding P-selectin glycoprotein ligand 1 (Marshall et al., 2003) and fibronectin binding integrin (Kong et al., 2009). Several studies have also shown that TCRs binding agonistic pMHC molecules can show catch bond behavior, whereas TCRs binding antagonistic pMHC molecules show slip bond behavior (Liu et al., 2014; Hong et al., 2015; Sibener et al., 2018). This would increase the difference in lifetime between different peptide ligands amplifying the power of discrimination of the agonistic TCR-pMHC interaction. Significant changes in the lifetime for the TCR-pMHC interaction typically take place already in the range of 0–10 pN (Liu et al., 2014; Sibener et al., 2018). Göhring et al. (2021) used a FRET force sensor to measure the force on 5c.c7 T cells binding either an anti-TCR single-chain variable fragment or the pMHC molecule IE^k^/MCC (Göhring et al., 2021). It was found that when the TCR binds an immobilized ligand the force applied on the TCR is significantly higher than when the TCR binds a ligand that is in a fluid membrane. This indicated that most of the force on the TCR-pMHC bond could be tangential to the bond direction, and that even low pN forces could rupture the bond between 5c.c7 TCR binding IE^k^/MCC (Göhring et al., 2021). Thus, this interaction behaved as a slip bond indicating that not all agonistic TCR-pMHC interactions are catch bonds. It could also explain why Huppa et al. observed an increase in the lifetime of this interaction upon disrupting the cytoskeleton (Huppa et al., 2010), an action that could be expected to reduce the force on the bond upon binding. Furthermore, Limozin et al. (2019) did only observe ideal and slip bonds for a range of different HLA-A2 binding 1G4 TCR, which is in line with that not all agonistic TCR-pMHC interactions have to show catch bond behavior (Limozin et al., 2019). This was obtained using the flow chamber method where 1G4 TCRs were immobilized on the channel surface and different HLA-A2 functionalized microspheres were introduced in the solution. Without force a lifetime in the range of 5–10 s was obtained, a value that for some studied TCR-pMHC interactions remained unaffected by forces up to 50 pN, indicative of ideal bonds (Limozin et al., 2019). For other interactions the force decreased the lifetime by a factor of 2–5 when applying 10–20 pN of force, indicative of slip bonds, but no catch bonds were observed.

In addition, it has been argued that catch bonds are a result of a geometric component and are only present when the force is exerted in a specific pulling geometry, as seen for the interaction between vinculin and F-actin (Huang et al., 2017). Another theory suggests that catch bonds could have been evolved to achieve higher stability of specific interactions to affect certain biological functions such as cell signaling and mechanosensing (Huse, 2017; Wang et al., 2019). In contrast, slip bonds may explain part of the underlying physical mechanism in cell migration through blood vessels, where bonds are continuously formed and broken between the surface of the vessel and a leukocyte (Alon et al., 1997). Other models focus on the importance of allosteric effects and of the sliding and rebinding of bonds under the influence of an external force (Lou et al., 2006; Lou and Zhu, 2007). Furthermore, measuring the full scope of how protein interactions respond to force requires varying the magnitude of the force, the loading rate, and the retraction speed. In addition, the mechanical properties of the transducer can affect the measured values which means that the choice of mechanical-based measuring techniques matters as well (Zhang et al., 2008).

## 10 Discussion

Despite past and current efforts to determine 2D binding kinetics of receptor-ligand complexes and connect them to their biological function, much remains to be done in this area. As discussed in the previous chapters, the lion´s share of binding kinetics so far is presented in 3D and converting these data to the more biologically relevant 2D binding kinetics is generally accepted to be non-trivial. In addition, it is found that the 2D binding kinetics are strongly dependent on local membrane-environmental factors. With this said, it is still striking that many studied ligand-receptor interactions in cell-SLB contacts have produced a characteristic confinement length, *h,* of around 1 nm, the length scale that relates 2D and 3D binding affinities according to Eq. 5. However, as illustrated in this review, there can still be significant differences in the obtained 2D kinetics from different studies. For example, lifetime measurements have argued that the 2D lifetime can be both significantly higher (Tolentino et al., 2008) as well as lower (Huang et al., 2010; Huppa et al., 2010) than those measured in 3D. In addition, other studies have shown similar values for 2D and 3D lifetimes (Robert et al., 2012; O’Donoghue et al., 2013). The latter is in agreement with the results from Bell on bond lifetimes where the forward and reverse rates from the encounter complex to the bound state is the same in 2D and 3D (Bell, 1978), whereas force applied on the bond or ligand rebinding can make the lifetime shorter or longer (Tolentino et al., 2008; Liu et al., 2014). Thus, differences in the methods and the systems used to study the 2D binding interactions can give rise to significantly different values. It is therefore of importance that the conditions under which the measurements are done are well controlled and defined in order to be able to compare results from different studies.

Generally, great care must be taken when choosing model system to ensure that the used method does not influence the parameters under investigation. Various aspects of a studied interaction can be assessed and the best suited method for this depends on the question asked. For example, measuring the interaction between single ligand-receptor bonds using micropipette-based manipulation of single cells has provided information about the affinity of early protein-protein binding events in cell-cell contacts (Huang et al., 2010; Liu et al., 2014), whereas fluorescence-based studies in cell-SLB contacts have given information about the affinity of binding events in already established contacts as well as what influence auxiliary binding molecules can have on these interactions (Dustin et al., 1997; Jönsson et al., 2016; Junghans et al., 2020). However, the binding affinity obtained with micropipette-based measurements is typically orders of magnitude lower compared to fluorescence-based studies, highlighting possible differences in binding between newly formed and stable contacts (Zhu et al., 2013). In addition, both theoretical studies, as well as studies on cell-free model systems, have shown a strong dependence of membrane fluctuations on the binding kinetics (Xu et al., 2015; Fenz et al., 2017; Steinkühler et al., 2018), whereas this behavior is less pronounced on living cells, if at all present (Hu et al., 2013; Junghans et al., 2020; Chouliara et al., 2021). Another factor that is often partly or fully omitted in model studies is the influence that the glycocalyx, i.e., the cell’s outer layer consisting of glycolipids and glycoproteins, has on binding kinetics. The glycocalyx will generally hide the ligands and receptors from each other on the meeting cells. To penetrate this the cell needs to apply a force in the form of protruding microvilli which are believed to form the first contact points between the meeting cells (Pettmann et al., 2018). However, cell contacts with an SLB form even when the cytoskeleton is disrupted using actin depolymerizing drugs such as latrunculin (Junghans et al., 2020). This indicates that other, microvilli independent, interactions can penetrate the glycocalyx barrier as well. Connected to this is that a minimum density of ligands is needed in order to initiate the formation of cell-SLB contacts as discussed in Chapter 6. This is particularly important when investigating weak binding events, since in this situation it is not certain that even moderate or high ligand densities will be sufficient to promote ligand binding without auxiliary binding molecules. Another factor that can influence the aforementioned parameters, and thus also the 2D binding kinetics of the studied interaction, is whether the cell is in a resting or activated state. It has been found that the 2D affinity of human CD2 binding CD58 (Zhu et al., 2006) and of TCR binding pMHC molecules (Huang et al., 2010) increased significantly upon T-cell activation. In the later study also the 2D lifetime was found to increase for activated vs naïve T cells (Huang et al., 2010). Care should thus be taken on the state of the T cells when measuring ligand-receptor affinity. This can in particular be a problem when using an SLB containing nickel-chelating lipids that on their own have been shown to induce cell activation unless effectively blocked (Dam et al., 2021). In order to minimize intracellular interactions upon cell activation, it is also possible to take advantage of protein design, for example by only expressing the extracellular region of the studied receptor on the cell surface (Junghans et al., 2020).

Although the current methods used to quantify 2D binding kinetics have provided essential data to better understand protein-protein interactions in cell contacts, there are still limitations with respect to measurements of weak affinities or short lifetimes. Since these interactions are stabilized *in vivo* by auxiliary binding molecules, techniques such as fluorescence-based methods that can take this into consideration are of special interest. However, due to the inherent spread in parameters such as receptor density among the cells, measuring weak affinities accurately involves averaging over a large number of cells, a time-consuming and often non-trivial approach. One way to circumvent this is to measure affinities on single cells (Chouliara et al., 2021), however, this has the potential drawback that the cell is in contact with the SLB for a longer time, possibly influencing the cell’s properties. In addition, due to exclusion of free ligands in the cell-SLB contacts, the effective density of ligands in the contact compared to outside the contact is often low, even below zero, for weak binding ligand-receptor pairs. This exclusion not only makes it more difficult to accurately delineate the cell-SLB contact but will also, if not properly corrected for, give rise to inaccurate affinity values. An alternative approach is to use single-protein tracking in the cell-SLB contact. Depending on the fraction of bound vs free ligands in the contact and the density of receptors on the cell this method could make it possible, in theory, to detect very weak binding events. One potential problem, however, is that weak binding events are typically associated with short lifetimes, which makes it complicated to discern free from bound molecules in the contact. Detecting lifetimes shorter than 100 ms is technically challenging using single-molecule tracking. However, interactions quicker than this are not unrealistic as even relatively strong interaction pairs, such as human CD2 binding CD58, have lifetimes in this regime when measured in 3D (Tolentino et al., 2008), and the CD4-pMHC class II interaction has been estimated to have a 2D lifetime of the order of 4 ms (Jönsson et al., 2016). New methods capable of obtaining accurate 2D lifetimes shorter than 100 ms would thus be of value.

Altogether, bioinspired membranes have made it possible to measure 2D binding kinetics for several key protein-protein interactions and have paved the way to better understand how various parameters influence this. Despite the continuous improvement of advanced imaging techniques, the complexity of cell-cell contacts *in vivo* is expected to remain a considerable obstacle for obtaining detailed biophysical information. Thus, the use of bioinspired membranes such as the SLB will likely continue to be a valuable and vital tool to understand cell adhesion and 2D binding kinetics for many years to come.
